# Replacing Broken Facings

**Published:** 1904-08

**Authors:** S. H. Gulford

**Affiliations:** Ex. Stomatologist.


### REPLACING BROKEN FACINGS.

An excellent and simple method of replacing broken facings in bridge work and crowns was shown by Dr. E. A. Bryant, of Washington, D. C. After removing any pieces of porcelain and the protruding pins from the old backing, a new facing is selected and ground to place, new holes being drilled to suit the new facing. The two holes in the backing are now united by drilling out the intervening metal, thus forming a slot. Wrapping the tooth with a single thickness of asbestos paper, and passing a piece of iron binding-wire around it to keep it in place, the pins which have been forced through the asbestos are now united by soft solder melted into place with a fine-pointed soldering-iron.

After trimming, so that the lug (formed of the pins and solder) will easily fit into the slot in the backing, and counter-sinking the slot on its lingual side, the flat surface of the facing and the lug are covered with good cement and forced into position. While the small amount of cement contained between two pins and pinholes will not prove sufficient to secure a new facing in position, the larger surface of the lug and the slot will retain enough to offer the desired resistance.

If preferred, when the cement has hardened, most of it may be removed from between the lug and slot, and its place supplied with amalgam, which readily unites with the soft solder.

The procedure is so simple and evidently so practical that it will probably be welcomed by many to whom the repair of crowns in the mouth has been a perplexing problem.

Another ingenious method in connection with crown work was the making of dowels to fit roots, no matter what their size or form may be.

It consisted in making long slender cones of thin platinum, shaping them to the root canal and then filling them with good solder. The pieces of platinum are cut in the shape of a V and folded around a fine tapering mandrel so that the edges lap considerably. A number are made of varying lengths and diameters. After the gold cap is made in the usual way to encircle and cover the end of the root, a hole is punched in its center and enlarged to the full size of the root canal. Through this hole one of the platinum cones (unsoldered so as to admit of shaping) is passed down into the canal as far as it will go. Into the opening cone a tapering piece of wood is passed and moved around so as to force the platinum into contact with the canal walls. The protruding portion of the cone is now burnished down upon the flat surface of the cap and the two united with hard wax. After removing from the root the cap and cone are invested and united with solder, enough being used to run down into the cone and entirely fill it.

After the crown is completed it may be set with gutta-percha, since making the dowel the same size and form of the canal but little of it will be needed, and hence the rigidity of the piece will not be impaired.—Dr. S. H. Gulford, Ex. Stomatologist.

